# Overnight Corticosterone and Gene Expression in Mouse Hippocampus: Time Course during Resting Period

**DOI:** 10.3390/ijms24032828

**Published:** 2023-02-01

**Authors:** Aneta Jaszczyk, Adrian M. Stankiewicz, Joanna Goscik, Alicja Majewska, Tadeusz Jezierski, Grzegorz R. Juszczak

**Affiliations:** 1 Department of Animal Behavior and Welfare, Institute of Genetics and Animal Biotechnology, Polish Academy of Sciences, 05-552 Jastrzebiec, Poland; 2 Department of Molecular Biology, Institute of Genetics and Animal Biotechnology, Polish Academy of Sciences, 05-552 Jastrzebiec, Poland; 3 Faculty of Computer Science, Bialystok University of Technology, 15-351 Białystok, Poland; 4 Department of Physiological Sciences, Institute of Veterinary Medicine, Warsaw University of Life Sciences (SGGW), 02-776 Warsaw, Poland

**Keywords:** glucocorticoids, corticosterone, brain, hippocampus, mice, transcriptomics, microarrays, gene expression, circadian cycle

## Abstract

The aim of the experiment was to test the effect of an elevated level of glucocorticoids on the mouse hippocampal transcriptome after 12 h of treatment with corticosterone that was administered during an active phase of the circadian cycle. Additionally, we also tested the circadian changes in gene expression and the decay time of transcriptomic response to corticosterone. Gene expression was analyzed using microarrays. Obtained results show that transcriptomic responses to glucocorticoids are heterogeneous in terms of the decay time with some genes displaying persistent changes in expression during 9 h of rest. We have also found a considerable overlap between genes regulated by corticosterone and genes implicated previously in stress response. The examples of such genes are *Acer2*, *Agt*, *Apod*, *Aqp4*, *Etnppl*, *Fabp7*, *Fam107a*, *Fjx1*, *Fmo2*, *Galnt15*, *Gjc2*, *Heph*, *Hes5*, *Htra1*, *Jdp2*, *Kif5a*, *Lfng*, *Lrg1*, *Mgp*, *Mt1*, *Pglyrp1*, *Pla2g3*, *Plin4*, *Pllp*, *Ptgds*, *Ptn*, *Slc2a1*, *Slco1c1*, *Sult1a1*, *Thbd* and *Txnip*. This indicates that the applied model is a useful tool for the investigation of mechanisms underlying the stress response.

## 1. Introduction

Glucocorticoids (GCs) are important in medicine for many different reasons. First, they constitute a crucial component of the stress response system [1] and are implicated in mechanisms underlying stress-related disorders such as posttraumatic stress disorder [2,3,4] and depression [5,6,7]. Second, glucocorticoids are commonly used in medicine due to their potent anti-inflammatory properties [8]. Finally, excessive levels of endogenous or exogenous glucocorticoids lead to Cushing’s syndrome, characterized by a set of metabolic, physiological, cognitive and psychiatric symptoms [9,10,11]. However, despite their importance in medicine, there are considerable gaps in our understanding of the mechanism mediating the effect of glucocorticoids on brain metabolism and physiology [12,13]. One of the most important problems is that previous experiments were focused on responses to glucocorticoids during the first 2 to 3 h after acute treatments while longer latencies are commonly neglected [12,13]. However, few available studies testing responses at multiple time points after treatment in astrocytic cell cultures [14] and liver [15] show that most of the transcriptomic responses to glucocorticoids appear at least 4 h–6 h or later after the treatment, which is consistent with delayed proteomic [15,16] and metabolic effects [17] peaking at about 7 h–12 h after the treatment. This delayed buildup of transcriptomic responses results from the fact that glucocorticoid receptors regulate the expression of numerous transcription factors and other regulatory molecules that lead to secondary effects [13]. The second issue is that the bulk of available transcriptomic data was obtained in in vitro cell cultures derived from perinatal brain tissue [13]. Such data are difficult to interpret because of profound physiological and metabolic differences between a developing and mature brain. The developing brain undergoes fast growth of cells governed by complex transcriptomic programs and specialized between-cell communication guiding the elongation of cellular protrusions and shaping connections between cells. In contrast, growth is severely restricted in the mature intact brain that specializes at this stage in the processing and integration of information and the complex regulation of transport between vascular and neuronal compartments. Additionally, the cell cultures are devoid of structure and components typical for the brain, including neurovascular units and distinct layers of cells having highly specialized local and long-distance connections with cells releasing various neurotransmitters. Finally, cell cultures are devoid of the contexts created by multiple hormonal systems scattered across the body and sleep-waking cycles orchestrating the functions of the entire organism. These methodological issues are further complicated by the problems inherent to past transcriptomic studies that are based on a small number of samples that were commonly pooled to decrease the costs of analyses [13,18]. As a result, available transcriptomic data suffer from low statistical power, leading to a large proportion of false positive and negative findings [13,18].

To fill the existing gaps in knowledge and to overcome limitations associated with past experiments, we designed an in vivo experiment to test the effect of corticosterone administered for 12 h during the period of circadian activity associated with the light–dark cycle. Furthermore, we also tested the dynamics of transcriptomic effects during the resting period when the level of corticosterone in mice returns to the baseline. As a result, we gained an insight into processes taking place in the brain after day-long stress or medication with glucocorticoids and during a subsequent resting period associated with the light–dark cycle. As far as we know, there are no other comparable studies. We also used relatively large groups (n = 8) that in combination with multiple time points and lack of pooled samples provide a large transcriptomic dataset (total n = 48) which is rarely encountered in transcriptomic studies. 

## 2. Results

### 2.1. Blood Corticosterone and Glucose

**Figure 1:**
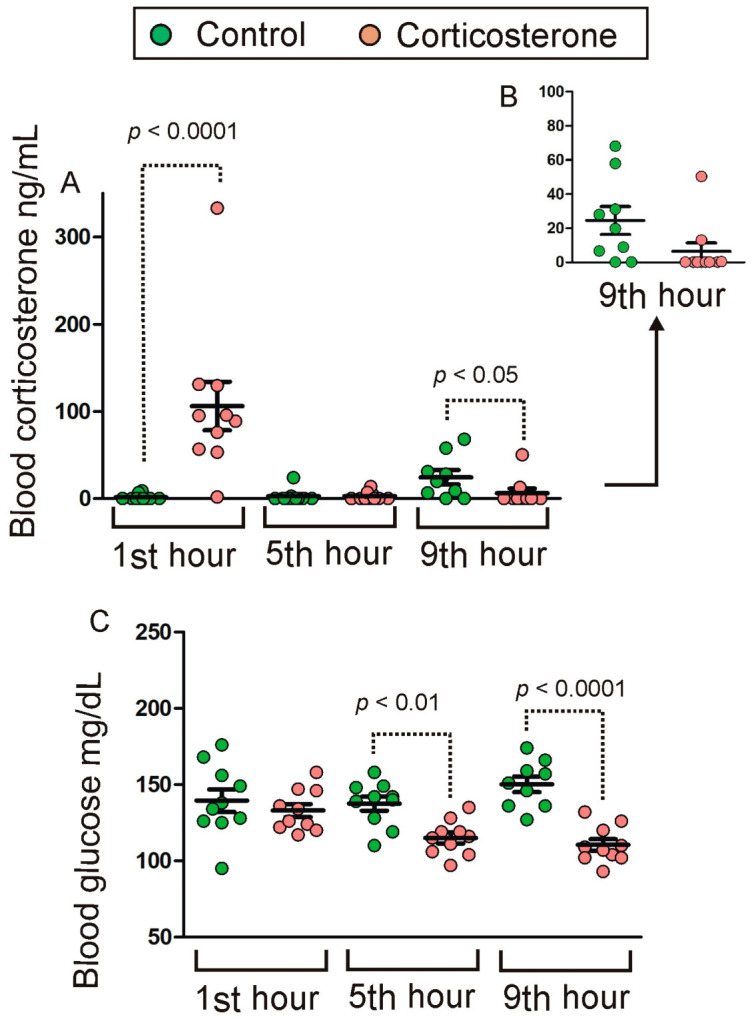
Effect of overnight corticosterone administered in drinking water on the blood level of corticosterone (**A**,**B**) and glucose (**C**) during the resting period occurring during the light phase. Data are presented as mean ± SEM overlayed on scatter plot of individual values. (**B**) part of data from panel (**A**) (ninth hour) shown with altered scale of Y axis.

Animals that received corticosterone in drinking water during the active period (dark phase) displayed an increased level of corticosterone at the beginning of the light phase (first hour/Figure 1A). The corticosterone returned to the baseline during the fifth hour of the resting period and remained at this level during the ninth hour in corticosterone-treated mice (Figure 1A,B). In contrast, control animals displayed a slight increase in corticosterone level during the last tested time point (ninth hour/Figure 1A,B). The data did not meet the requirement of variance homogeneity and, therefore, were analyzed with a nonparametric Mann–Whitney *U* test that showed significant differences between corticosterone-treated and control mice during the first (U = 2, n1 = n2 = 10, *p* = 0.0003) and ninth hour (U = 20, n1 = 9, n2 = 10, *p* = 0.04) and a lack of differences during the fifth hour of the resting period (U = 45, n1 = n2 = 10, *p* = 0.7). Differences between groups in the level of blood corticosterone were not associated with differences in water usage that includes both amounts of ingested water and spillage during the course of the experiment. The Mann–Whitney *U* test showed the lack of significant differences in water usage between control and corticosterone-treated mice during the first (U = 28, n1 = n2 = 10, *p* = 0.096), fifth (U = 27.5, n1 = n2 = 10, *p* = 0.089) and ninth (U = 42, n1 = 9, n2 = 10, *p* = 0.81) hour.

The blood level of glucose in corticosterone-treated and control mice (Figure 1C) was similar during the first hour of the resting period but displayed a gradual decrease in corticosterone-treated mice during subsequent time points (fifth and ninth hour). ANOVA revealed a significant effect of treatment [F(1,53) = 31.59, *p* < 0.0001] and a significant interaction between treatment and time of sample collection [F(2,53) = 5.49, *p* = 0.007] with significant differences between corticosterone and control groups during the fifth (*p* = 0.002) and ninth hour (*p* < 0.0001) of the resting period as indicated by the post hoc Fisher’s LSD test.

### 2.2. PCR Validation of Microarray Results

**Figure 2:**
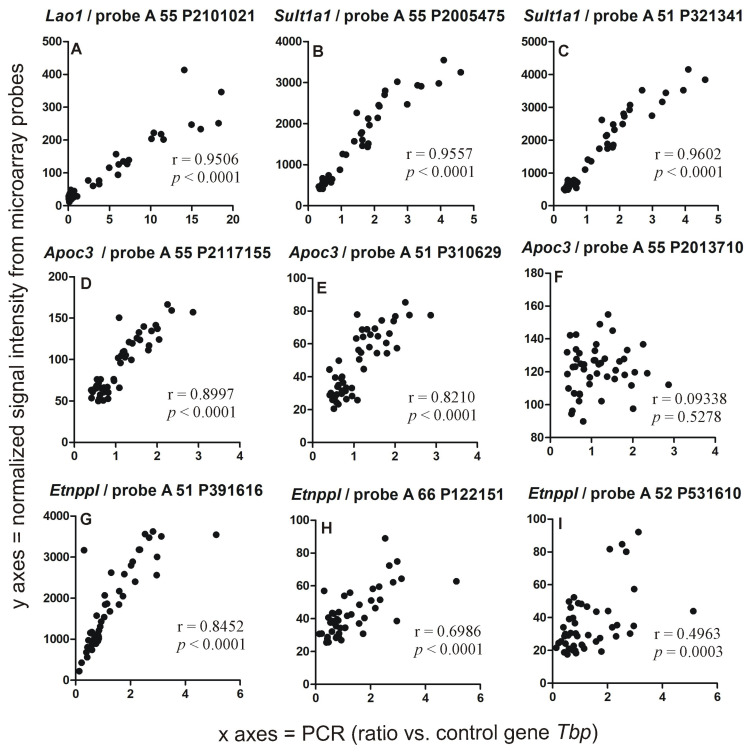
Correlation between results obtained with PCR and microarrays calculated separately for each probe annotated to tested genes *Lao1* (**A**), *Sult1a1* (**B**,**C**), *Apoc3* (**D**–**F**) and *Etnppl* (**G**–**I**). The microarray signal was normalized and decomposed into single channels as described in the methods section. r—Pearson’s correlation coefficient.

**Figure 3:**
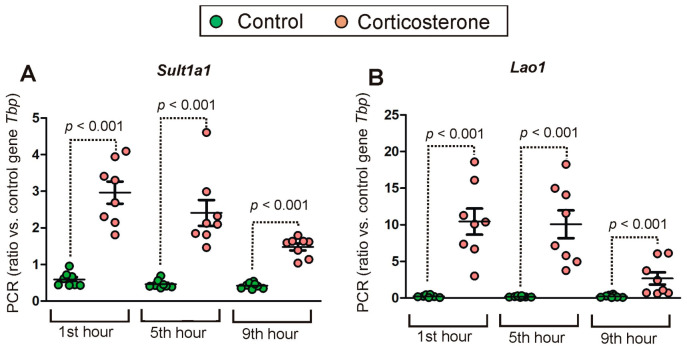
Expression pattern of *Sult1a1* (**A**) and *Lao1* (**B**) revealed by PCR. Data are presented as mean ± SEM overlayed on scatter plot of individual values. *p* values were determined based on Mann–Whitney U test.

Initially, we selected six genes (*Sult1a1*, *Lao1*, *Etnppl*, *Apoc3*, *Plin4* and *Pla2g3*) for validation but for two of them (*Plin4* and *Pla2g3*) we were not able to design proper starters because they yielded additional products. Therefore, the final validation was performed for *Sult1a1*, *Lao1*, *Etnppl* and *Apoc3*. While the selection of genes was based on significant effects observed for specific probes: A 55 P2117155 (*Apoc3*), A 51 P391616 (*Etnppl*), A 55 P2101021 (*Lao1*), A 55 P2005475 (*Sult1a1*) and A 51 P321341 (*Sult1a1*), the results of PCR analysis were compared with all microarray probes annotated to selected genes (Figure 2). The calculation of correlations shows high congruence between PCR results and initially selected microarray probes (Figure 2A–D,G) indicating that microarrays reliably detected a level of validated genes. Similar conclusions are drawn from between-group comparisons. The PCR analysis showed increased expression of *Sult1a1* and *Lao1* in all tested time points (Figure 3) with a *p*-value < 0.001 as indicated by the Mann–Whitney *U* test (U = 1, n1 = n2 = 8) and the same pattern of expression has been found in microarray data (File S1).

**Figure 4:**
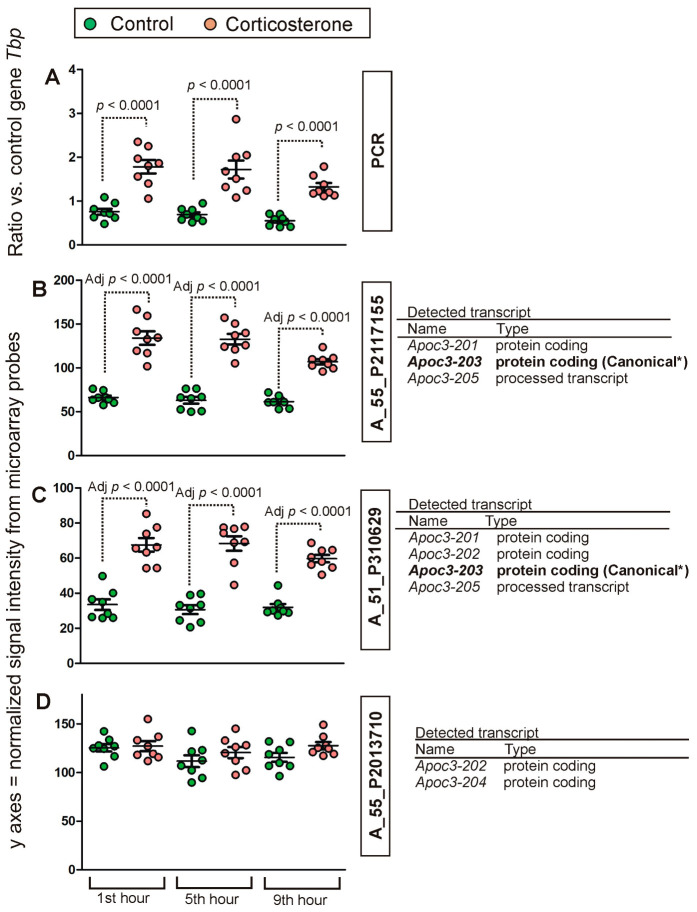
Between-group comparison of *Apoc3* expression detected with PCR (**A**) and different microarray probes (**B**–**D**). *p* values were determined based on LSD test of PCR results (**A**) and separate channel test applied for microarray data (**B**,**C**). The right panel provides information about transcript variants (Ensembl/BioMart database) that can be detected by the probes. *—Ensembl canonical transcript having the highest coverage of conserved exons, highest expression, longest coding sequence and represented in other key resources, such as NCBI and UniProt. Definitions of transcript types are provided in File S2.

Additionally, the analysis shows variability between individual probes in their ability to detect the expression of annotated genes. These differences were most striking in the case of the gene *Apoc3* (Figure 2D–F). Comparisons between groups (File S1) showed that the two best probes for detecting *Apoc3* (determined on the basis of correlation with PCR results/Figure 2D,E) detected similar changes between control and corticosterone-treated animals (Figure 4B,C). This finding is consistent with PCR results (Figure 4A) that revealed a significant effect of treatment [F(1,42) = 129.78.49, *p* < 0.0001] with significant differences between corticosterone and treatment groups during all tested time points (*p* < 0.0001, Fisher’s LSD test). In contrast, the third probe that was not correlated with the PCR results (Figure 2F) failed to detect the effect of treatment (Figure 4D). To understand better the differences between probes that were initially annotated to the same gene, we retrieved additional information on detected transcripts from the Ensembl/BioMart database. These data revealed that the probes detect various variants of the *Apoc3* transcript and that the best-correlated probes (Figure 2D,E) share the ability to detect the Ensembl canonical transcript (Figure 4B,C) defined as a variant having the highest coverage of conserved exons, highest expression, longest coding sequence and represented in other key resources.

**Figure 5:**
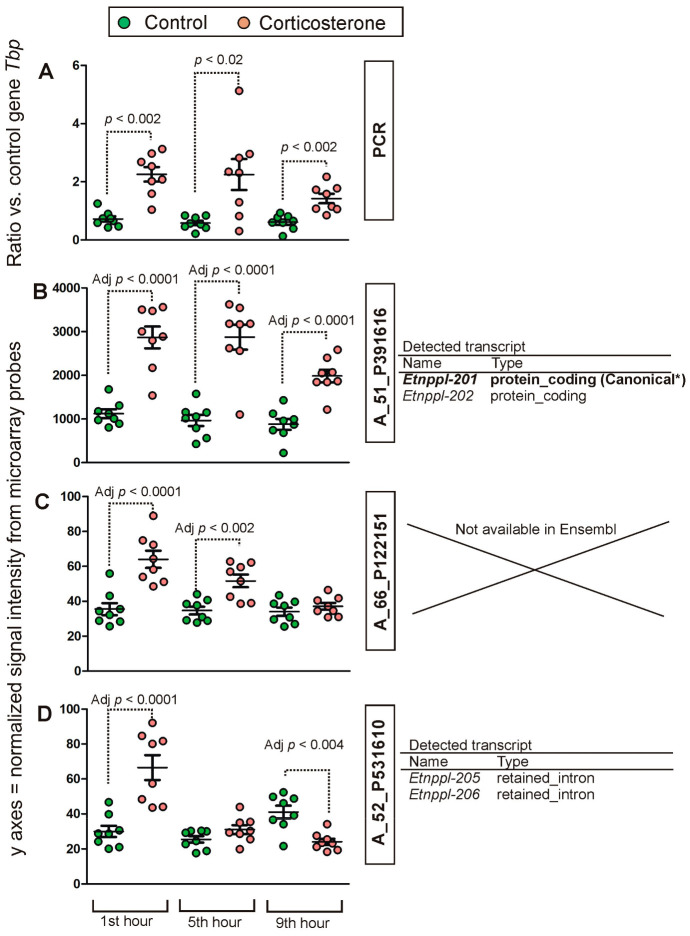
Between-group comparison of *Etnppl* expression detected with PCR (**A**) and different microarray probes (**B**–**D**). *p* values were determined based on Mann–Whitney U test applied to microarray PCR results (**A**) and separate channel test applied to microarray data (**B**,**C**). The right panel provides information about transcript variants (Ensembl/BioMart database) that can be detected by the probes. *—Ensembl canonical transcript having the highest coverage of conserved exons, highest expression, longest coding sequence and represented in other key resources, such as NCBI and UniProt. Definitions of transcript types are provided in File S2.

Even more complex patterns emerged in the case of the second gene (*Etnppl*) detected by multiple probes that provided highly discrepant results in terms of correlation with PCR (Figure 2G–I). Between-group comparisons of PCR data (Mann–Whitney *U* test) showed significant differences during the first (U = 1, n1 = n2 = 8, *p* = 0.001), fifth (U = 9, n1 = n2 = 8, *p* = 0.016) and ninth hour of the resting period (U = 1, n1 = n2 = 8, *p* = 0.001) (Figure 5A). Between-group comparisons of microarray data (File S1) showed that one probe detected an increased expression in all three time points (Figure 5B), consistent with PCR data. The second probe detected increases in two time points (Figure 5C). Finally, the last one detected increased expression in the first time point and an opposite effect in the last time point (Figure 5D) following, in fact, changes in the level of the blood corticosterone level (Figure 1A). Additionally, these probes displayed considerable differences in signal intensity (Figure 5B–D). Data retrieved from the Ensembl/BioMart database showed that the probe detecting the Ensembl canonical transcript (Figure 5B) provided results displaying the highest correlation with the PCR while the lowest correlation was obtained in the case of the probe detecting only alternatively spliced transcripts considered to contain intronic sequences (Figure 5D). These results prompted us to retrieve Ensembl/BioMart data for all other probes available in this database.

### 2.3. Transcriptomic Changes in Control Animals during the Resting Period

**Figure 6:**
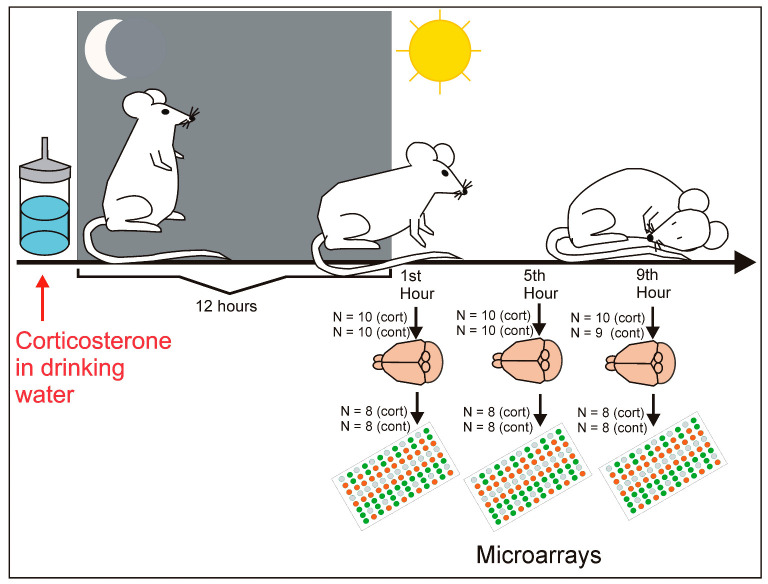
The design of the experiment. Mice received either corticosterone solution or vehicle at the end of the light phase, followed by 12 h of dark phase which is a period of mouse activity. The next day, the animals were sacrificed at three time points to collect brain samples for transcriptomic analysis performed with microarrays. For more details please see Section 4.2.

Circadian rhythms in gene expression are not the main objective of this study but provide a crucial context for changes in the expression of GC-responsive genes during the resting period (Figure 6). Therefore, we compared the transcriptome in control animals during consecutive time points of the light phase. The largest effect was found in the comparison between the first and the last time points (first hour vs. ninth hour) because 312 unique microarray probes displayed significant differences with an adjusted *p*-value < 0.05, and this comparison detected most of the significant findings (File S3). In contrast, only three microarray probes displayed significant differences in the comparison between the first and the second time points (first hour vs. fifth hour) while 11 probes differed between the second and the third time points (fifth hour vs. ninth hour). These additional comparisons revealed a few additional genes (adjusted *p* < 0.05) that were only specific for the comparison between the first and fifth hour (*Slc15a3*) or between the fifth and ninth hour (*Hrk* and *Cables1*).

**Figure 7:**
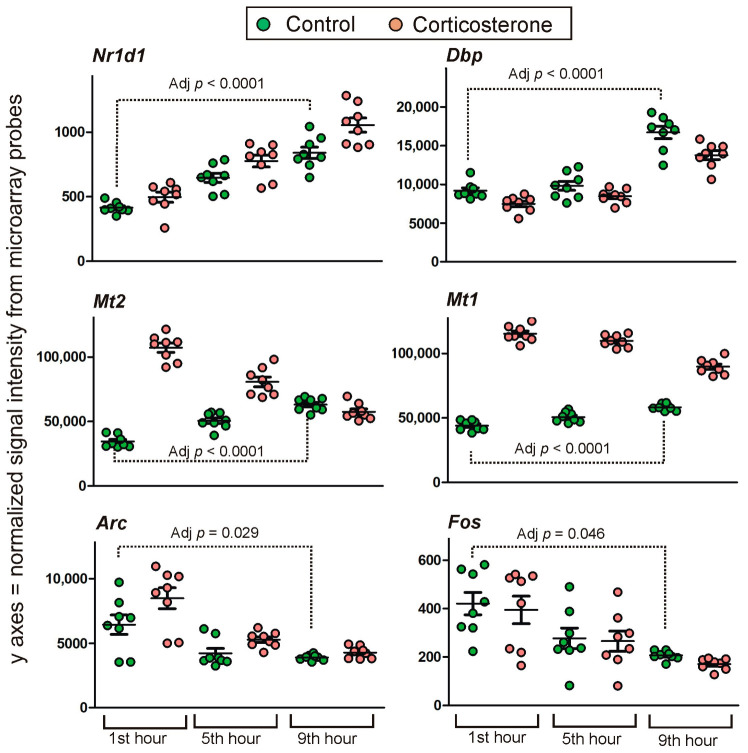
Selected genes displaying significant changes in control animals during the resting period. Y axis indicates probe signal intensity decomposed into green and red channel after final background correction, within-array and between-array normalization. All data are available in File S3.

The 312 microarray probes displaying significant differences during the longest testing interval (first hour vs. ninth hour) included 11 probes that failed the annotation, two probes that were inconsistently annotated depending on the applied method, and 10 probes that can detect more than one gene. Data available in BioMart indicate that according to currently applied models almost 90% of probes displaying a time-dependent effect detect protein-coding transcripts, while the remaining 10% of probes detect lncRNAs (5.8%) or transcripts considered as dysfunctional (nonsense-mediated decay, retained intron, protein coding loss-of-function variants, processed transcript and processed pseudogene). Importantly, the time-dependent changes in gene expression included genes known to be involved in the regulation of circadian rhythms such as *Nr1d1*, *Dbp*, *Ciart* (*Gm129*), *Arc* and *Fos* [19,20,21,22] confirming a pattern typical for the resting period in rodents. Examples of genes that changed the expression in control animals during the resting period are shown in Figure 7.

### 2.4. Effect of Corticosterone—General Characteristics of Microarray Results

**Figure 8:**
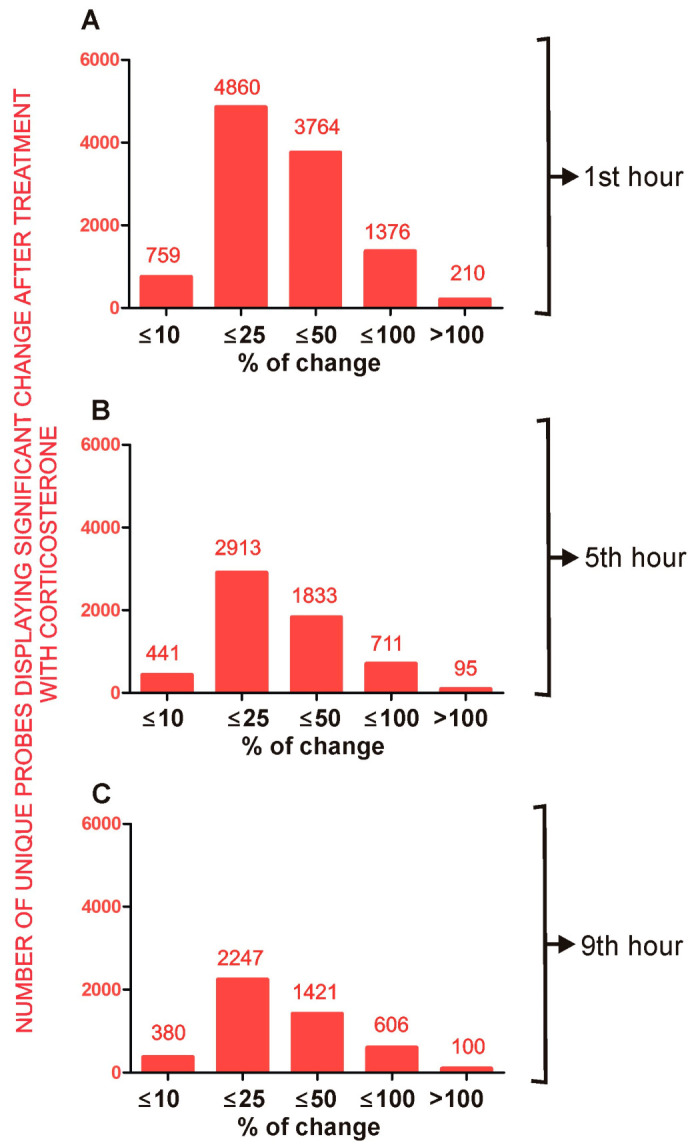
General characteristics of primary effects of corticosterone that are significant at least during the first hour of the resting period. (**A**)—number of unique probes that indicated significant differences during the first hour of the resting period. (**B**)—number of unique probes that indicated significant differences during the first and the fifth hour of the resting period. (**C**)—number of unique probes that indicated significant differences during the first, fifth and ninth hour of the resting period. Numbers above the bars indicate an exact number of unique probes showing significant differences between groups separately for categories based on the magnitude of altered expression. All data are available in File S1.

**Figure 9:**
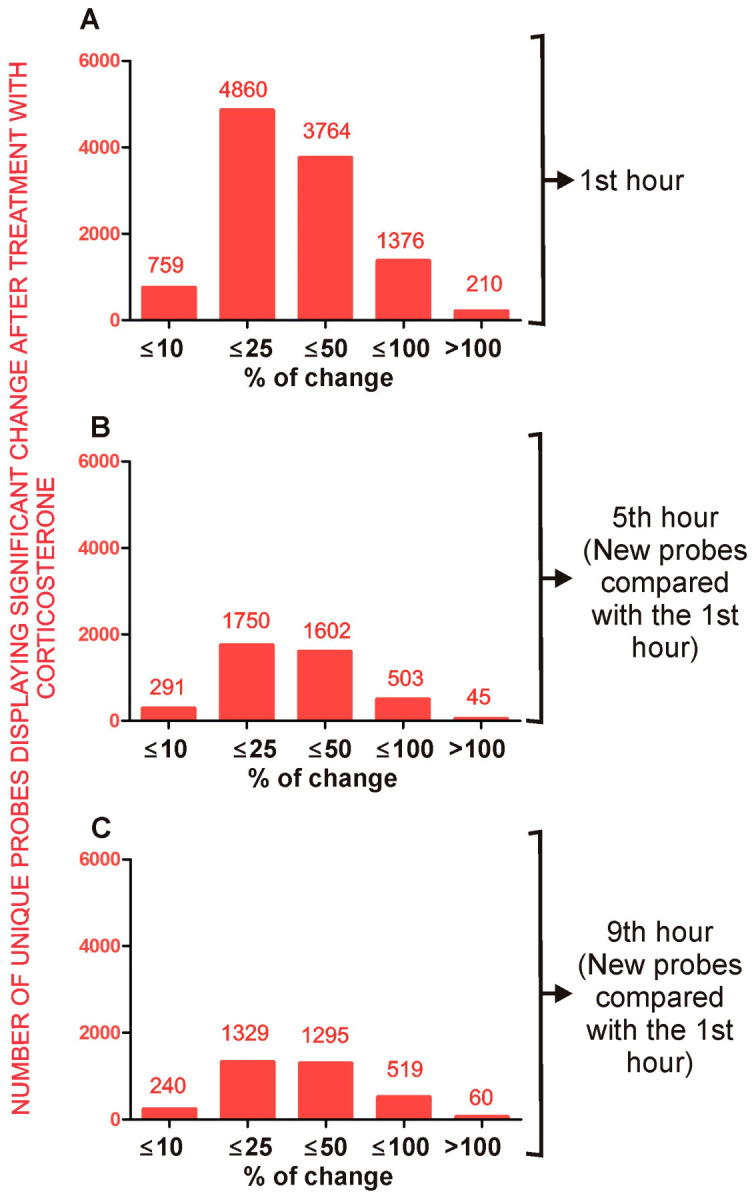
General characteristics of secondary effects of corticosterone characterized by significant differences occurring only during the fifth and/or the ninth hour of the resting period (**B**,**C**) in comparison with primary effects shown in panel (**A**). (**A**)—number of unique probes that indicated significant differences during the first hour of the resting period representing primary effects. (**B**)—number of unique probes that indicated the lack of effect of corticosterone during the first hour and significant differences during the fifth hour of the resting period. (**C**)—number of unique probes that indicated the lack of effect of corticosterone during the first hour and significant differences during the ninth hour of the resting period. Numbers above the bars indicate an exact number of unique probes showing significant differences between groups separately for categories based on the magnitude of altered expression. All data are available in File S1.

The statistical analysis revealed significant changes between corticosterone-treated and control animals in all three tested time points (File S1). In total, 17,444 unique probes indicated significant differences between groups during at least one tested time point while the remaining 39,161 probes provided only insignificant results. To differentiate gross regulatory mechanisms, we divided the microarray results into primary effects (10,969 unique probes/Figure 8) that were already significant at the time of an elevated level of corticosterone (the first hour of the light phase) and secondary effects (6475 unique probes/Figure 9) that became significant during the fifth and seventh hour when the level of corticosterone returned to the baseline in corticosterone-treated animals.

#### 2.4.1. Primary Effects

The comparison between corticosterone-treated and control animals sacrificed during the first hour (Figure 8A) revealed almost 11,000 unique probes that differed between groups although half of them (51.2%) displayed small changes that were not larger than 25% (absolute value of log2 fold change ≤ 0.32). In contrast, there were 3764 probes differing within the range of 25% and 50% (absolute value of log2 fold change > 0.32 and ≤0.58), 1376 probes differing within the range of 50% and 100% (absolute value of log2 fold change > 0.58 and ≤1) and only 210 probes displaying differences larger than 100% (absolute value of log2 fold change > 1). The primary effects decreased over time. After 9 h of rest, the number of probes displaying a significant effect of corticosterone decreased to 43% of all significant effects observed during the first hour (Figure 8C).

##### Long-Lasting Primary Effects

Some primary effects were maintained for the entire testing period (first, fifth and ninth hour) despite the fact that elevated levels of glucocorticoids were observed only during the first hour in corticosterone-treated mice compared with the control group. In total, 3670 probes displayed significant differences during all three tested time points with the same direction of changes between groups. Some microarray probes were not specific to single genes according to the BioMart/Ensembl database (145 probes) or were inconsistently annotated by different databases (74 probes). After the removal of such probes, there were 3451 probes specifically annotated to 3144 genes. Most of the genes code proteins although there were also lncRNAs (154), miRNA (1) lincRNA (2), rRNA (1), TEC (To be Experimentally Confirmed) genes (14), 169 pseudogenes (4.9%) and 96 dysfunctional transcripts (2.8%) classified in the Ensembl database as processed transcripts, transcripts retaining introns, nonsense mediated decay and antisense transcripts (definitions are provided in File S2).

**Figure 10:**
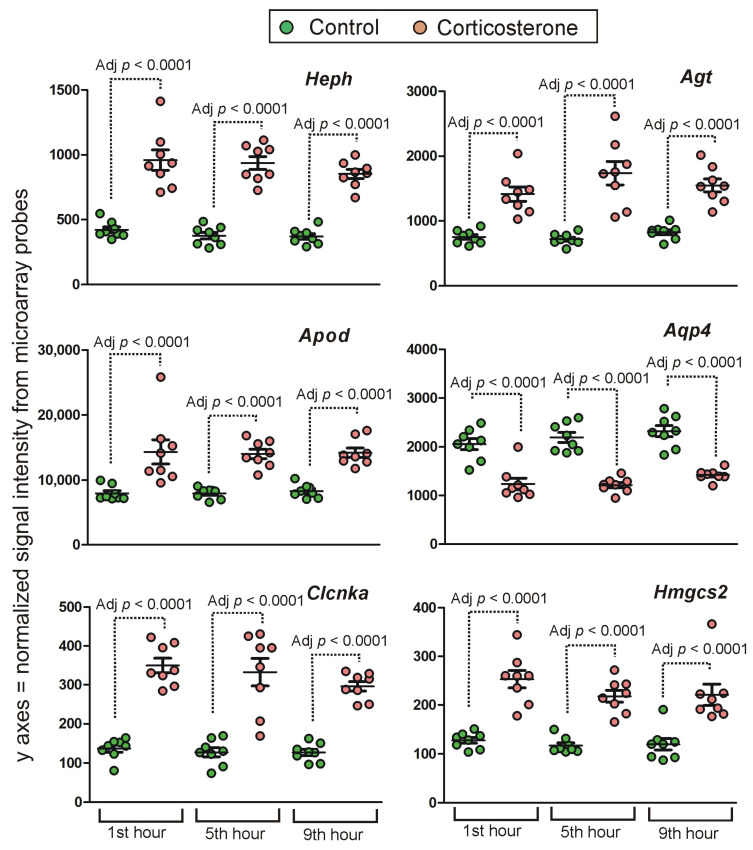
Examples of genes displaying long-lasting primary effects. Y axis indicates probe signal intensity decomposed into green and red channel after final background correction, within-array and between-array normalization. All data are available in File S1.

The majority of probes (2991) indicated small (<25%) and medium (<50%) differences between groups. Differences larger than 100% (log2 fold change > 1) after 9 h of rest were indicated by only 77 probes annotated to 71 genes. Furthermore, the majority of them displayed very low signal intensity (mean < 50). After the rejection of these probes, the group was restricted to seven protein-coding genes (*Etnppl*, *Sult1a1*, *Heph*, *Pygm*, *Pla2g3*, *Clcnka* and *Lao1*). It should be noted that in the case of *Etnppl* (Figure 5), *Pygm*, *Pla2g3* and *Heph* (File S1) the effect was specific for some variants of the transcripts. Importantly, a prolonged expression of *Etnppl*, *Pygm*, *Pla2g3* and *Heph* was detected by probes binding canonical variants of transcripts. Smaller but still considerable differences (the range between 50 and 100% after 9 h of rest) were indicated by 383 probes annotated to 327 genes (File S1). This group was also dominated by probes with small signal intensity (mean < 50). After their rejection, the group was restricted to only 22 genes (*Mt1*, *Ptgds*, *Apod*, *Fam107a*, *Timp4*, *Phyhd1*, *Aqp4*, *Pxmp2*, *Hmgcs2*, *Agt*, *Pygm*, *Plin4*, *Vmn1r48*, *Kansl3*, *Rgs12*, *Opalin*, *Smim4*, *Col5a3*, *Apoc3*, *Ugt1a6b*, *Olfr145*, *Gm10447*). In the case of *Opalin*, the effect was detected by a probe-binding transcript variant with retained intronic sequences. The examples of genes displaying the most persistent changes in expression during all tested time points are shown in Figure 10.

##### Intermediate Primary Effects

**Figure 11:**
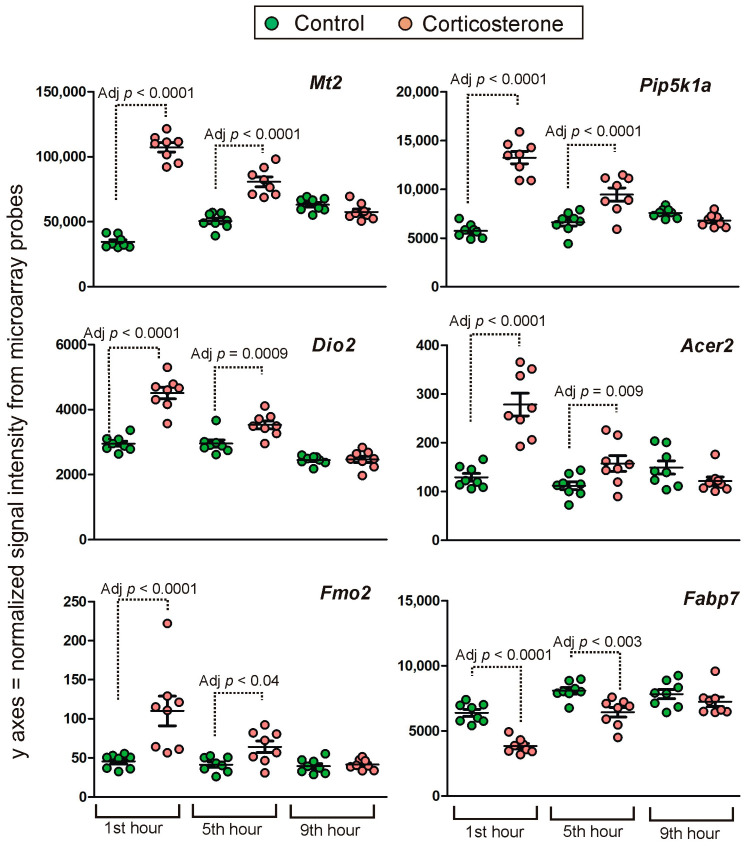
Examples of genes displaying intermediate primary effects. Y axis indicates probe signal intensity decomposed into green and red channel after final background correction, within-array and between-array normalization. All data are available in File S1.

A number of 2301 unique probes displayed significant differences during the first and the fifth hour with the same direction of changes during both tested time points but returned to the basal level during the ninth hour of the resting period. Examples of this pattern of expression are shown in Figure 11. As already noted (Figure 5), there is variability in the pattern of expression detected by probes binding different variants of transcript derived from the same gene. To restrict the data to genes that display altered expression exclusively up to the fifth hour, we excluded 248 probes indicating primary effects that were significant at a longer interval (ninth hour). Some remaining microarray probes were inconsistently annotated by different databases (34 probes) or were not specific to single genes according to the BioMart/Ensembl database (63 probes). After their removal, there were 1956 probes specifically annotated to 1781 genes. Most of them code proteins although there are also lncRNAs (77), miRNA (1) lincRNA (1), 12 TEC genes (To be Experimentally Confirmed), 2 unknown but likely coding genes, 41 (2.1%) pseudogenes and 52 (2.7%) dysfunctional transcripts, classified in the Ensembl database as processed transcripts, transcripts retaining introns, antisense, nonsense-mediated decay and LoF transcripts (definitions are provided in File S2). The majority of probes (1737) indicated small (<25%) and medium (<50%) differences between groups. Differences larger than 100% (log2 fold change > 1) after 5 h of rest were indicated by only 11 probes annotated to 11 genes but most of them displayed very low signal intensity (mean < 50). After the rejection of these probes, the group was restricted to two protein-coding genes (*Cdkn1a* and *Maff*). In the case of *Cdkn1a* (File S1), the effect was specific for some variants of the transcripts. Importantly, highly significant changes occurring during the first and fifth hour were detected by a probe binding canonical variant of *Cdkn1a* transcripts. Smaller but still considerable differences (the range between 50 and 100% after 5 h of rest) were indicated by 208 probes annotated to 181 genes (File S1). This group was also dominated by probes with small signal intensity (mean < 50). After their rejection, the group was restricted to only 26 genes (*8430426J06Rik*, *Alpl*, *Atp2c2*, *Ccl12*, *Cmtm2a*, *Cntfr*, *Ecscr*, *Fmo2*, *Gbp2*, *Gbp3*, *Gjb6*, *Glp2r*, *Gm12022*, *Gm4285*, *Lrg1*, *Map3k6*, *Mt2*, *Ninj2*, *P2ry12*, *Pdpn*, *Sdc4*, *Slc38a5*, *Stab2*, *Tekt4*, *Tmem52* and *Tmprss6*).

##### Short-Lasting Primary Effects

**Figure 12:**
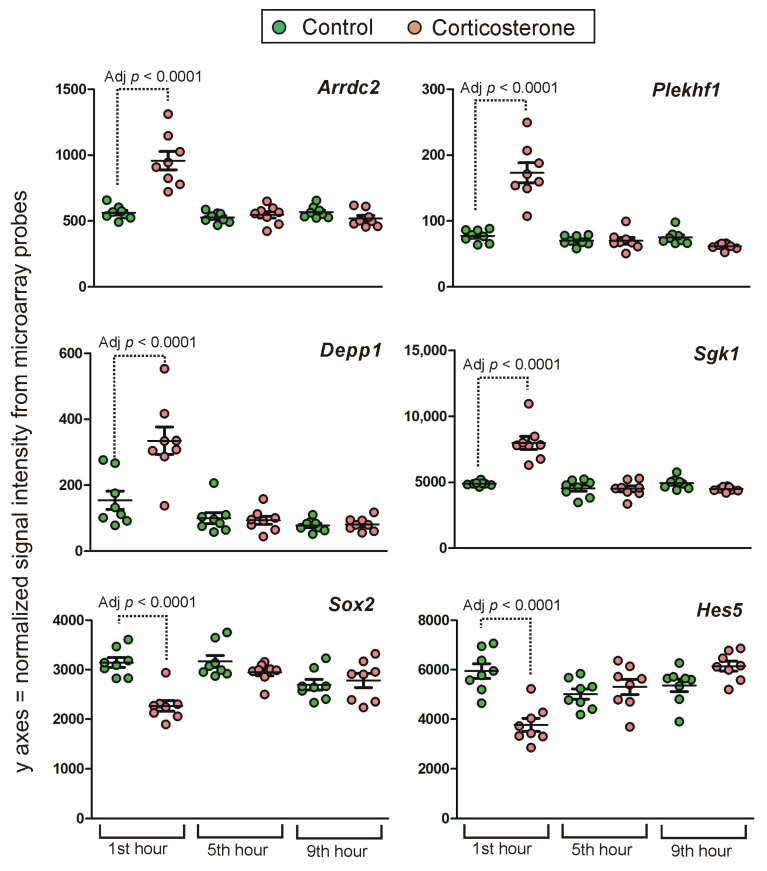
Examples of genes displaying short-lasting primary effects. Y axis indicates probe signal intensity decomposed into green and red channel after final background correction, within-array and between-array normalization. All data are available in File S1.

A number of 3911 probes indicated differences between groups only during the first hour of the resting period which was associated with an increased level of glucocorticoids in animals receiving exogenous corticosterone. Examples of this expression pattern are shown in Figure 12. Transcription leads to the occurrence of different variants of transcripts derived from the same gene and thus probes annotated to the same gene may provide a discrepant result (Figure 5). Therefore, we have removed 916 probes to restrict the data to genes that displayed differences only during the first hour of the resting period. Additionally, some microarray probes were not specific to single genes according to the BioMart database (75 probes) or were inconsistently annotated by different databases (27 probes). After the removal of these probes, there were 2893 probes specifically annotated to 2490 genes. Most of them code proteins although there are also lncRNAs (198), miRNAs (2) lincRNAs (2), snRNA (1), 26 TEC genes (To be Experimentally Confirmed), 71 (2.5%) pseudogenes and 74 (2.6%) dysfunctional transcripts classified in the Ensembl database as processed transcripts, transcripts retaining introns, antisense, nonsense-mediated decay and LoF transcripts (definitions are provided in File S2).

The majority of probes (2490) indicated small (<25%) and medium (<50%) differences between groups. Differences larger than 100% (log2 fold change > 1) were indicated by only 33 probes but most of them displayed very low signal intensity (mean < 50). After the rejection of these probes, the group was restricted to six genes (*Kcnq2*, *Depp1*, *Galnt15*, *Plekhf1*, *Cxcl10* and *Phactr3*). In the case of *Kcnq2*, *Cxcl10* and *Phactr3* (File S1) the effect was specific for some variants of the transcripts but only in the case of *Cxcl10* the significant effect was detected by a probe binding canonical variants of the transcripts. The most perplexing case is the *Kcnq2* gene because a significant effect was detected by a probe binding only dysfunctional variants of the *Kcnq2* transcripts (retained intron and processed transcript lacking an open reading frame) while five other probes indicated a lack of differences between groups. Smaller but still considerable differences (the range between 50 and 100% after 5 h of rest) were indicated by 370 probes (File S1). This group was also dominated by probes with small signal intensity (mean < 50). After their rejection, the group was restricted to only 20 genes (*Hes5*, *Sgk1*, *Mgp*, *Fzd2*, *Arrdc2*, *Pdk4*, *Vgll3*, *Thbs4*, *Rtp4*, *Gata2*, *Ifit3b*, *Tnfsf10*, *Cytl1*, *Tcim*, *BC018473*, *A330032P22Rik*, *Phf11d*, *Lhx3*, *BC053393* and *Acss3*).

##### Time-Dependent Reversal of Primary Effects

**Figure 13:**
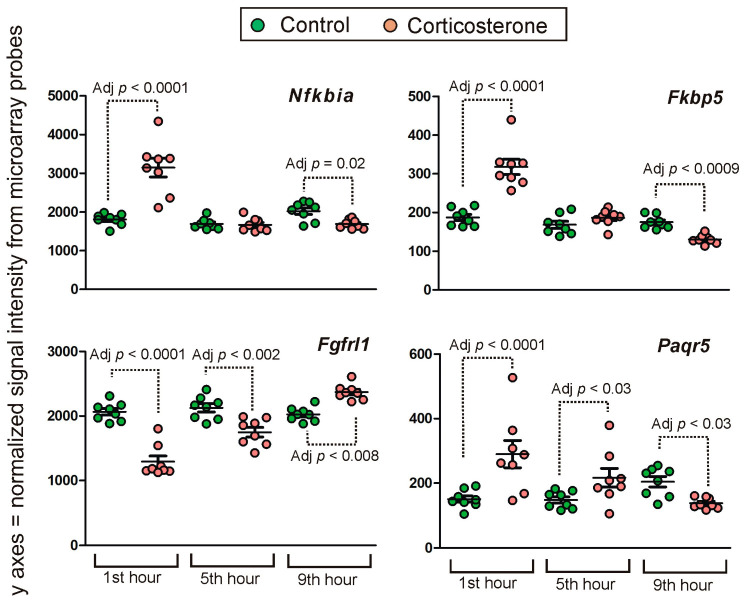
Examples of genes displaying time-dependent reversal of primary effects. Y axis indicates probe signal intensity decomposed into green and red channel after final background correction, within-array and between-array normalization. All data are available in File S1.

A relatively small number of corticosterone-responsive genes significantly reversed the direction of expression during the resting period. Examples of this expression pattern are shown in Figure 13. There were 75 probes indicating a reversal in expression and most of them detect protein-coding genes with exception of one lncRNA, two processed pseudogenes and one transcript containing intron. Additionally, some probes are inconsistently annotated in different databases (two probes) or are annotated to more than one gene in the Ensembl/BioMart database (two probes). After the removal of probes that are lacking specificity, we found 71 genes that reversed the expression including 39 genes that were unique for the category of primary transcriptomic responses with the time-dependent reversal. The remaining 32 genes also displayed other expression patterns detected by additional probes. All unique 39 genes displayed signal intensity larger than 80 and the largest differences between groups (of more than 50%) were found in the case of seven protein-coding genes (*Paqr5*, *Fas*, *Nfkbia*, *Fkbp5*, *Fgfrl1*, *Mc4r*, *Smim3*).

#### 2.4.2. Secondary Effects

One of our assumptions was that the transcriptomic changes induced during an elevated level of corticosterone (first hour) will trigger a second wave of transcriptomic effects that will develop when corticosterone returns to the baseline. However, the secondary effects were smaller in terms of the number of probes, indicating significant differences only during the fifth and ninth hour of the resting period (Figure 9B,C) compared with the first hour (Figure 9A). Furthermore, although there were 60 probes indicating changes larger than 100% after 9 h of rest, all of them were characterized by small signal intensity (mean < 50). Very low signal intensity was also found in the case of probes indicating differences in the range between 50 and 100% after 9 h of rest because most of them (99%) were characterized by very low signal intensity (mean < 50). After the rejection of probes with the lowest signal intensity, the group was restricted to only five genes (*Zbtb16*, *Sh3pxd2b*, *Rhcg*, *Asb4*, *LOC102635912*, *Gjb3* and *Gipc2*).

## 3. Discussion

### 3.1. Characteristics of Experimental Model

From a methodological point of view, the most important findings are corticosterone data (Figure 1A) because they confirm the effectiveness of our experimental procedure. The experimental design allowed both for a significant increase in blood corticosterone level and its normalization during the resting period. Therefore, this procedure enabled us not only to study transcriptomic responses to an elevated level of glucocorticoids but also to determine the time course of their decay and to identify persistent effects that remain despite the return of glucocorticoids to the baseline. This information is important to better understand the prolonged effects of glucocorticoids during circadian rhythms of activity and rest. The corticosterone data also show that exogenous glucocorticoids administered in drinking water inhibited the release of endogenous corticosterone consistently with the well-known regulatory mechanisms [23,24]. This effect is visible in corticosterone-treated mice as a decrease in blood corticosterone in the afternoon compared with control mice that display a small increase in blood corticosterone (Figure 1A,B) consistently with physiological fluctuations of glucocorticoid release [25]. It should be noted, however, that we could observe only an early stage of the rising level of endogenous corticosterone because the last tested time point in the afternoon (ninth hour) was about 3–4 h before the peak of endogenous corticosterone secretion. The afternoon increase in the blood level of corticosterone in resting control animals is associated with the increase in the level of glucose (Figure 1C) which is in accordance with the role of glucocorticoids in the regulation of glucose homeostasis [12]. However, we have not observed such an effect during the first hour of the resting period that was associated with a highly elevated level of glucocorticoids in corticosterone-treated mice. Such a finding may seem to be surprising but in fact is consistent with experiments showing that an acute effect of glucocorticoids on blood glucose in mice is present in fasted animals but not in animals having free access to food [26]. Therefore, the detection of an altered level of glucose was most likely in our experiment after several hours of rest when animals were sleeping but not immediately after the end of the active phase associated with food consumption. Our observations indicate that during the first hour after turning on the lights (the first hour of the resting period), mice are still awake but their activity is declining compared with the dark period. In contrast, during later periods of the light phase mice are laying motionless with closed eyes indicating sleeping. We were not directly monitoring sleep in this experiment because of technical limitations (EEG) and because the most important factor for us was the return of blood corticosterone level to the baseline. This was sufficient for drawing conclusions about the persistency of transcriptomic responses to exogenously applied glucocorticoids. However, some insights can be provided by a pattern of expression of genes associated with the regulation of the circadian rhythms (Figure 7) such as *Nr1d1*, *Dbp*, *Ciart* (*Gm129*), *Arc* and *Fos* [19,20,21,22]. These data indicate an undisturbed circadian rhythm and provide a context for the interpretation of transcriptomic responses to corticosterone.

### 3.2. General Characteristics of Transcriptomic Response to Corticosterone

The transcriptomic response to overnight corticosterone treatment was dominated by primary effects (Figure 8) that were present at the time of an elevated level of blood corticosterone during the first hour of the resting period (Figure 1A). These effects involved much more probes (Figure 9A) than secondary effects that occurred later during the resting period (Figure 9B,C). The primary effects waned over time although some of them persisted even after 9 h of rest (Figure 8C) despite the fact that corticosterone returned to the baseline. This indicated that transcriptomic responses to corticosterone are heterogeneous in terms of the decay latency and that some of them may contribute to the long-lasting effects of glucocorticoids. It is also worth noting that the time course of decay depends on the probe used for the detection of individual genes (Figure 4 and Figure 5). Some probes annotated to the same gene provide almost identical results (Figure 2B,C) while other probes give discrepant results (Figure 2D–I). Some light is shed by the data retrieved from the BioMart/Ensembl database showing that probes annotated to the same gene are frequently detecting different variants of transcripts (Figure 4 and Figure 5) including both the most representative canonical variants and a number of dysfunctional variants. This indicates that each unique probe should be considered separately even if several probes are annotated to the same gene. This approach provides opportunities to better understand patterns of the expression of individual genes but also creates challenges in the interpretation of large-scale datasets. Unfortunately, not all microarray probes are available in the BioMart/Ensembl database leading to numerous gaps in data concerning transcript variants.

The second problem with the interpretation of transcriptomic data is a large number of significant results with small changes between groups (Figure 8 and Figure 9). Therefore, it is important to understand both biological and methodological mechanisms leading to the occurrence of such results. A small magnitude of detected changes may result from genuine differences between groups. For example, transcriptomic changes restricted to a small population of highly specialized cells are diluted in the total pool of transcripts isolated from homogenized tissue [18] and such a scenario is especially likely to occur in the brain that is highly heterogeneous in terms of cells [27]. Furthermore, the collection of samples may be performed at the early or late stages of gene regulation when the observed changes are small. Our results support such a hypothesis because some well-known GC-responsive genes such as *Errfi1*, *Klf9*, *Bcl6* that are responsive to the acute administration of glucocorticoids [13] display significant but small differences (<30%) after the overnight administration of corticosterone. On the other hand, small changes in detected expression may constitute systematic errors generated by background correction and array normalization. Therefore, we should assume that the lower the magnitude of detected effects, the higher probability of false positive findings in transcriptomic data. Nonetheless, there is no perfect method allowing for the separation of genuine effects from technical errors in a single study. This problem can be overcome, however, by a meta-analytic approach that allows for the identification of replicable findings and their separation from random effects in pooled datasets derived from different studies [18]. This approach depends, however, on the availability of data and is negatively affected by the selective publishing of transcriptomic results [18]. Therefore, we publish all significant results in supplementary data although the main focus of the paper is genes displaying the most conspicuous differences between groups.

### 3.3. Time-Course of Transcriptomic Responses to Corticosterone during the Resting Period

Our study shows that transcriptomic responses to glucocorticoids are heterogeneous in terms of the decay time (Figure 10, Figure 11, Figure 12 and Figure 13). Importantly, the number of transcriptomic responses that display short-term duration or even time-dependent reversal during the resting period (*Errfi1*, *Cdkn1a*/*p21*, *Ddit4*/*Redd1*, *Ndrg2*, *Sesn1*, *Wnt7a*) are involved in the negative control of cell growth and proliferation [13,28,29,30,31,32,33,34,35]. It is known that acute stress triggers widespread activation affecting 96% of the brain [36]. Therefore, stress-induced inhibition of cell growth and proliferation is considered an adaptive mechanism protecting the brain from the adverse effects of excessive excitation including the genotoxic action of reactive oxygen species and the redundant tropic effect of glutamate [18]. However, prolonged inhibition of trophic processes can adversely affect cognitive processes that depend on neurogenesis and neuronal plasticity. Our results suggest that GC-induced impairment in cell growth and proliferation in mice is prone to recovery during resting periods associated with low levels of glucocorticoids. This is especially important in the case of patients suffering from post-traumatic stress disorder (PTSD) that display a decreased brain volume [37] and typically suffer from sleep disturbances such as insomnia and nightmares [38,39,40]. Furthermore, our findings support the therapeutic approach of applying a treatment of sleep impairments as a crucial step in the treatment of PTSD [40]. It should be noted, however, that molecular processes taking place in the human brain are very poorly investigated and, therefore, we do not know whether processes observed in rodent brains are comparable with human biology.

On the other hand, the number of GC-responsive genes including some core genes displayed persistent changes in expression during the entire resting period, despite a quickly normalized level of blood corticosterone. Such prolonged effects may result from persistent changes in methylation leading to altered accessibility of chromatin or from very slow degradation of some transcripts. The case of *Etnppl* gene (Figure 5) suggests that some prolonged effects may indeed result from the slow rate of transcript degradation. This is because there was a prolonged increase in the level of the dominant variant of the *Etnppl* transcript (Figure 5B), while variants with retained intronic sequences displayed a short-term increase in expression that was followed by a reversal of differences (Figure 5D) corresponding to the level of the blood corticosterone (Figure 1). In this case, the immature or aberrant transcripts with intronic sequences are likely to indicate the rate of transcription while a dominant variant may represent the rate of transcript degradation. Obviously, this is only a hypothesis and the precise mechanism underlying prolonged changes in transcript levels should be verified experimentally. It should also be noted that not all data concerning the variants of transcripts are easy to interpret. For example, prolonged changes in the expression of *Opalin* during all tested time points were only detected by a probe binding transcript variant with retained intron but not by a probe expected to bind canonical variant of this gene according to the BioMart/Ensembl database. This indicates that despite considerable progress, there is still uncertainty about the properties of some probes and/or bioinformatic models used to predict the properties of different variants of transcripts.

Importantly, persistent transcriptomic responses occurring during the resting period indicate long-lasting processes affected by glucocorticoids. Inspection of the most affected genes that differed more than 50% after 9 h of rest indicates that GCs can induce long-lasting effects including the metabolism of lipids (*Etnppl*, *Apod* and *Pla2g3* [41,42,43]), ketones (*Hmgcs2* [44]) and glycogen (*Pygm* [45]), homeostasis of iron (*Heph* [46]), water and potassium (*Aqp4* [47]), blood pressure (*Agt*), peroxisomal transport (*Pxmp2* [48]), actin dynamics (*Fam107a* [49]), inhibition of tissue remodeling (*Timp4* [50]), epigenetic regulation (*Kansl3* [51]), voltage-sensitive chloride channels (*Clcnka* [52]) and, finally, removal of toxins and signaling molecules (*Ugt1a6b*, *Sult1a1* and *Mt1* [53,54,55,56,57]). Some of these genes also induce pleiotropic effects. For example, *Ptgds* (*L-PGDS*) is responsible for the synthesis of prostaglandin D2 regulating a wide range of processes such as vasodilation, immune responses and sleep homeostasis [58]. The functions of affected genes are consistent with a broad range of effects induced by glucocorticoids including the metabolism of lipids, glycogen and iron [12,13], the immune response [59] and cardiovascular system [60,61].

Our study suggests that persistent changes in gene expression constitute an important mechanism of the delayed effects of glucocorticoids. The second mechanism of delayed effect is the reversal of expression during the resting period due to the inhibition of the endogenous release of corticosterone. In our study, there were relatively few genes reversing expression but it should be noted that we tested expression at an early stage of the rising level of corticosterone. However, endogenous corticosterone achieves the highest level at the beginning of the active period [25]. Therefore, differences between control and corticosterone-treated mice can increase over time leading to a larger number of genes reversing expression due to inhibition of the release of endogenous glucocorticoids in corticosterone-treated animals. Finally, the third mechanism of delayed effects of glucocorticoids may involve changes in the expression of genes that are indirectly regulated by glucocorticoids due to changes in the expression of various transcription factors [13]. Our study suggests that these secondary responses play a minor role during the resting period. Importantly, although a considerable number of probes indicated secondary changes larger than 50% (Figure 9B,C), the signal intensity obtained from the majority of these probes is very low. Such signal can be provided by a small number of cells with distinct patterns of gene expression compared with the majority of cells associated with the central nervous system. Potentially, the source of such genes can be various blood cells trapped in the dissected tissue. Therefore, the significance of these findings for brain physiology is uncertain.

### 3.4. Comparison with the List of Established GC-Responsive Genes

The comparison between transcriptomic data derived from different studies constitutes a special challenge because of frequent changes in gene nomenclature and inconsistencies between different databases used for the annotation of microarray probes [18,62]. Additionally, a common problem encountered in transcriptomic studies is a low statistical power due to small groups and pooling of samples leading to a large number of false positive and negative findings in individual studies [18,63,64,65]. Finally, the comparability between studies is decreased by the selective publishing of transcriptomic data [18]. As a result, individual studies available in the literature contain a mixture of true positive, false positive and false negative findings. To avoid this problem, we compared our current results with a referential list of GC-responsive genes that is based on the meta-analysis of standardized data retrieved from 17 studies [13] and includes a most recent update of gene nomenclature [18]. The referential dataset [13] is based both on in vivo [66,67,68,69,70,71,72,73] and in vitro experiments [14,74,75,76,77,78,79,80,81] and is dominated by acute data obtained during the period ranging from 1 to 6 h after administration of glucocorticoids. Based on these literature data, we created a list of the most frequently and consistently reported genes that were additionally divided into core and extended parts differing in the number of supporting studies. The core list contains 88 most frequently and consistently regulated genes that displayed the same direction of change in at least four papers [13,18] while an extended list contains 251 genes that displayed the same direction of change in three independent studies in response to glucocorticoids [18].

In our present experiment, we found significant changes in the expression of 69 (78%) core genes and 188 (75%) GC-responsive genes from an extended list (assignment to already established GC-responsive genes is provided in File S1). Therefore, we have found most of the expected genes indicated already in the literature data [13]. The changes in expression of the core GC-responsive genes displayed several patterns. Some of these genes (*Cdo1*, *Ddit4*, *Ehd3*, *Fzd1*, *Lyve1*, *Mtmr2*, *Nedd9*, *Pdk4*, *Plekhf1*, *Rhou*, *Sesn1*, *Sgk1*, *Sox2*, *Tle4*, *Tmem109*, *Wnt7a*, *Zfp36l1*) displayed significant differences only during the first hour of rest, which was associated with an elevated level of corticosterone, and returned to baseline during the rest of the experimental period. The second group was altered during the first hour, maintained significant differences during the fifth hour of rest and returned to the baseline during the ninth hour of rest (*Gjb6*, *Klf15*, *Mertk*, *Mt2*, *Ndrg2*, *Pim3*, *Prr5*, *Rasl11b*, *Rhob*, *Sdc4*). Additionally, a few genes (*Cdkn1a* and *Svil*) could be classified either to the first or second group depending on the probe used for their detection. The third group of genes (*Fkbp5* and *Nfkbia*) reversed the direction of expression at the end of the experiment following a similar effect on the level of blood corticosterone. The fifth group (*Arl4d*, *Azin1*, *Calm2*, *Chst1*, *Lhfp*, *Ppp5c*, *Rdx*, *Sult1a1*) displayed significant differences during all three-time points with the same direction of expression. Finally, the remaining core genes displayed a more complex pattern of expression that was frequently probe-specific. While the involvement of these genes in the response to GC is well established [13], the time-course of their expression during the resting period was not previously reported. Our study, which is based on a large number of independent samples, also indicates that the previous list of most replicable glucocorticoid-responsive genes which was based mostly on acute effects [13] should be extended with a special emphasis on genes that are regulated at longer intervals such as 8–12 h. Especially striking findings are genes displaying a replicable pattern of expression during two and three independent time points with a high magnitude of detected changes after 12 h of treatment that were not previously implicated in the glucocorticoid response [13]. Genes such as *Pip5k1a*, *Pmaip1*, *Gbp3*, *Tekt4*, *Gm11627*, *Maff*, *Ddc*, *Pnpla2*, *Pglyrp1*, *Alpl*, *Slc38a5*, *Lao1*, *Etnppl*, *Clank*, *Heph*, *Phyhd1*, *Timp4*, *Agt*, *Timp4*, *Vmn1r48*, *Pdzd2*, *Pygm*, *Apod*, *Serpinb1a*, *Crybb1*, and *Tfcp2l1* belong to this group. Therefore, these data will constitute an important contribution to an update of our previous meta-analysis [13] that is scheduled after the publication of the remaining data from our ongoing glucocorticoid project.

### 3.5. Comparison with Transcriptomic Response to Stress

An important question is to what extent the effects induced by exogenous corticosterone overlap with effects observed during the stress response that are much more complex than just a release of glucocorticoids and involves other components such as vascular effects [18,82,83] and the release of neurotransmitters [84,85]. In addition, neurotransmitters and glucocorticoids not only induce their specific effects on gene expression [13,84,85] but also interact with each other [12,86,87]. Therefore, not all effects observed after the administration of corticosterone may be relevant to the stress response. Therefore, we compared the corticosterone results with the referential list of stress-responsive genes [18]. The comparison showed that 1702 GC-responsive genes are also reliably detected in experiments testing the effect of stress on brain transcriptome (File S1). This indicates that GCs can contribute up to 63.7% percent of transcriptomic responses observed during the stress response and this estimate is much higher than the previous one based predominantly on acute responses obtained during the period ranging from 1 to 6 h after administration of glucocorticoids [18]. In the group of transcriptomic responses common for GCs and the stress response are genes displaying the most persistent changes during the resting period such as *Etnppl*, *Heph*, *Fam107a*, *Apod*, *Aqp4*, *Agt*, *Ptgds*, *Mt1*, *Plin4*, *Sult1a1* and *Pla2g3*. Importantly, we have also found some genes that were not previously implicated in the glucocorticoid response [13] but were found to be top genes in the stress response [18] such as *Depp1*, *Galnt15*, *Mgp*, *Hes5*, *Txnip*, *Il1r1* and *Elovl7*, and in the case of short-term primary effects, *Slc2a1*, *Acer2*, *Fabp7*, *Pglyrp1*, *Lrg1*, *Htra1*, *Fmo2*, *Htra1*, *Gjc2*, *Lfng*, *Thbd*, *Jdp2*, *Slco1c1*, *Fjx1*, *Pllp* in the case of intermediate primary effects and *Opalin*, *Mobp*, *Slc4a4*, *Tmem88b*, *Trf*, *Ptn*, *Actb*, *Qk*, *Homer1*, *Junb*, *Ptn*, *Creb5* and *Kif5a* (long-lasting primary effects). This indicates that the applied model of overnight corticosterone treatment is a useful tool for studying mechanisms underlying the stress response.

## 4. Materials and Methods

### 4.1. Animals

Sixty Swiss-Webster male mice (weighing 39.0 ± 3.8 g (mean ± SD) and 12 weeks of age) were used in the experiment. Mice were obtained from a breeding colony located at the Institute of Genetics and Animal Biotechnology (Jastrzebiec, Poland). Animals were housed in cages with fine sawdust bedding (4–5 mice per cage) under standard conditions (12/12 h light cycle, 22 ± 2 °C, and 55 ± 5% humidity). The animals had an enriched environment and free access to dry food (Labofeed H, Kcynia, Poland) and tap water. The experiment was performed with the permission of the Second Local Ethical Committee in Warsaw (permission number: WAW2/090/2018) in accordance with the Polish Animal Protection Law of 15 January 2015 on the protection of animals used for scientific and educational purposes.

### 4.2. Experimental Procedure

Three-month-old mice were relocated from family cages to individual cages and, next, were moved to the experimental room dedicated only to this experiment in order to limit the human presence and activity that could disturb animals. The separation into individual cages was performed for two major reasons. First, to enable a selection of individual mice for tissue collection without disturbing other animals. Second, to avoid antagonistic behaviors that frequently occur in group-housed male mice that develop a social hierarchy with dominant and subordinate littermates [88,89]. After the separation, the mice were divided into control and corticosterone-treated groups. The mice assigned to the corticosterone-treated group were divided randomly into three subgroups (n = 10). For each corticosterone group a separate control group (n = 10) of siblings was assigned so that the obtained results could be compared between brothers from both groups. Each group contained animals from five different litters. Although the initial number of animals was 10 in each group, the final number of animals decreased to nine in one of the control groups (Figure 6) because we noticed a tumor of salivary glands in one of the mice during the later stage of the experiment. Single-housed animals were left undisturbed for 21 days to habituate them to the new conditions following the procedure used previously in our laboratory [82,83]. The habituation is performed because mice separated into individual cages display an increased reactivity to environmental stimuli that returns to the baseline after about 3 weeks [83] together with a lowered level of corticosterone [90].

The main part of the experiment started on day 22 when half of the animals received corticosterone dissolved in drinking water (100 µg/mL) with the addition of hydroxypropyl-β-cyclodextrin (0.45%) which is a cyclic oligosaccharide used to dissolve steroid hormones in water [91,92]. Corticosterone was initially dissolved in a 30% solution of hydroxypropyl-β-cyclodextrin with the help of a vortex/magnetic stirring bar and was diluted to obtain the final concentration. The dose of corticosterone was set on the basis of previous studies [93,94] and additional pilot experiments. Both literature data [93] and our results confirmed that detected levels of corticosterone are within the range observed under physiological conditions after stress in mice [95,96]. Control mice received only water with the addition of hydroxypropyl-β-cyclodextrin (0.45%). Mice received new bottles with either corticosterone solution (22 mL) or vehicle (22 mL) at the end of the light phase, followed by 12 h of dark phase which is a period of mouse activity. Additionally, the bottles were weighed before and after the experiment to control water utilization including the amount of water ingested by animals and spillage caused by the manipulation of bottles by experimenters and the activity of animals. These control measurements showed that all animals had a sufficient amount of available water. The next day, the animals were sacrificed at three time points to collect samples for analysis (Figure 6). The first group of corticosterone-treated mice and assigned control subjects were sacrificed during the first hour of the light phase when animals are still awake although their activity was declining. The remaining corticosterone-treated and control mice were sacrificed during the fifth and ninith hour, i.e., during the resting phase. Cages with animals intended for sample collection were quietly transferred from the experimental room to the adjacent dissection room immediately before the sacrifice was performed by cervical dislocation followed by decapitation. Animals from the control and corticosterone groups were sacrificed in alternating order. Trunk blood was collected for corticosterone and glucose assessment while brains were removed for hippocampal dissection performed according to the protocol described previously [97]. Dissected whole hippocampi were placed in freezing vials, then frozen in liquid nitrogen and stored at a temperature of −80 °C. 

### 4.3. Analysis of Blood Samples

Blood was collected in Eppendorf tubes containing 20 µL of 0.4 mM Na_2_EDTA. Next, 1 µL of blood was used to assess the level of glucose with a Microdot glucometer (Cambridge Sensor USA, Plainfield, USA ) and dedicated test strips (9–10 biological replicates per group and two technical replicates per mouse). The remaining blood was centrifuged (10 min/5000 RPM at +4 °C) to collect plasma that was stored at −20 °C. The plasma corticosterone level was checked by an enzyme-linked immunosorbent assay (Demeditec Corticosterone rat/mouse ELISA kit). One sample was replicated twice on the plate. It means that there were 9–10 biological replicates per group and two technical replicates per mouse. The test was performed according to the protocol provided by the manufacturer, and the absorbance for each well was read at 450 nm.

### 4.4. RNA Isolation

The total RNA was extracted from the individual hippocampal samples using GeneMATRIX universal RNA purification kit (EURx Ltd., Gdansk, Poland) following the protocol provided by the manufacturer. The quantity and quality of all RNA samples were assessed by Nanodrop ND-1000 spectrophotometer (Thermo Fisher Scientific, Waltham, MA, USA) and Bioanalyzer 2000 microcapillary electrophoresis (Agilent Technologies, Santa Clara, CA, USA). High-quality samples (260/280~2.1, RIN > 9) were next selected for the microarray analysis (n = 8 in each group). 

### 4.5. Microarrays

The analysis of the gene-expression profile was performed using SurePrint G3 Mouse Gene Expression v2 8 × 60 K Microarray, 8 × 60 K (Agilent Technologies, Santa Clara, CA, USA) and Agilent Technologies Reagent Set according to the manufacturer’s instructions. The characteristics of microarrays are provided in Table 1. RNA Spike In Kit (Agilent Technologies, USA) was used as an internal control, the Low Input Quick Amp Labeling Kit was applied to amplify and label (Cy3 or Cy5) target RNA to generate complementary RNA (cRNA) for oligo-microarrays; 300 ng of cRNA from control (Cy3-labelled) and corticosterone-treated (Cy5-labelled) mice were hybridized together on two-color microarrays without pooling samples from the same groups. In total, we used 24 microarrays printed on three slides, with eight microarrays applied for each time point. It means that there were eight biological replicates per group and one technical replicate per mouse. Both control and corticosterone-treated animals from all analyzed time points were assigned to each slide in a pseudo-random way. The Gene Expression Hybridization Kit was used for fragmentation and hybridization and the Gene Expression Wash Buffer Kit was used for washing slides after hybridization. The acquisition and analysis of hybridization intensities were performed using an Agilent DNA Microarray Scanner G2505C. Data were extracted and the background subtracted using the standard procedures included in the Agilent Feature Extraction Software version 10.7.3.1. Data extraction included Lowess normalization. The data were deposited in the GEO database (accession number GSE218508). 

### 4.6. Annotation of Microarray Data

Due to the variability between different genomic databases [62,98], we have applied consensus annotation [98] combining two different annotation approaches. The first annotation consisted of the following steps. Each probe was annotated with a gene symbol list using biomaRt R package with “agilent sureprint g3 ge 8 × 60 k” attribute [99], GPL21163-3202.txt annotation file from the GEO database [100] and GPL21163_noParents.an.txt annotation file from the gemma database [101]. If no gene symbol existed, the probe sequence was annotated with Ensembl identifiers using the rBLAST R package [Basic local alignment search tool, https://github.com/mhahsler/rBLAST, accessed on 1 April 2022] followed by the translation of these identifiers to gene symbols using BioMart. The second annotation was based on BioMart/Enseble database and combined data from mouse and mouse strain databases (version 107) since some probes are included only in the mouse strain databases. BioMart/Ensembl does not contain the most recent version of the Agilent mouse microarrays that were used in our experiments (v2 8 × 60 K). Therefore, we combined data retrieved for 8 × 60 K and WholeGenome agilent microarrays. In the retrieved BioMart/Ensembl annotation dataset, we included information about the gene name and type, gene description, transcript name and type and assignment to the Ensembl canonical category of transcripts having the highest coverage of conserved exons, highest expression, longest coding sequence and represented in other key resources (https://www.ensembl.org/info/genome/genebuild/canonical.html, accessed on 19 July 2022). Finally, we compared the first and the second annotation to identify consistently annotated probes, including gene synonyms retrieved from BioMart/Ensembl with the term “gene name” selected in the filter panel and the terms “gene name” and “synonyms” selected in the attributes panel. 

### 4.7. Microarray Data Analysis

The raw data files were analyzed with the Limma package from the Bioconductor project using the same criteria for all files [102]. The ‘normexp’ background correction method [103] has been applied. The background correction was followed by within-array normalization carried out with the loess procedure and between-array normalization was conducted with the quantile method [104,105]. Normalized data without offset were used for the calculation of fold changes and retrieval of separate channel intensities from M (binary logarithm of red/green intensity ratio) and A (average log_2_ intensity of the microarray spot) values with the following formulas:

$$Red\;channel=\sqrt{2^{2\;\cdot \;A\;value}\cdot 2^{M\;value}}$$

$$Green\;channel=\frac{Red\;channel}{2^{M\;value}}$$

Data with offset 50 (variance stabilizing transformation) were used for the calculation of *p* values following previous guidelines [103,106]. The statistical analysis was performed with separate channel tests which take into consideration the intra-spot correlation [107]. *p*-values were corrected using the Benjamini and Hochberg procedure controlling False Discovery Rate [108]. Genes showing adjusted *p*-values < 0.05 were considered differentially expressed. 

### 4.8. PCR Validation 

We have selected six genes (*Sult1a1*, *Lao1*, *Etnppl*, *Apoc3*, *Plin4* and *Pla2g3*) for validation with RealTime qPCR based on the statistical analysis of microarray data and our interests in the function of individual genes. Primers were designed using the Primer-BLAST tool (https://www.ncbi.nlm.nih.gov/tools/primer-blast/, accessed on 14 June 2021). Primers included all mRNA transcripts of each gene and were located on two different exons. The verification of primers was performed by temperature gradient PCR (55–65 °C), followed by gel electrophoresis. We were not able to design proper starters for two genes (*Plin4* and *Pla2g3)* because they yielded additional products. Therefore, these genes were excluded after we failed to design two sets of primers for each of these two genes. The reference gene *Tbp* was selected using NormFinder v0.953 software (https://moma.dk/normfinder-software [109], accessed on 14 June 2021) from a group of four candidate genes (*Hmbs*, *Ywhz*, *Tbp* and *Gapdh*). The specification of primers used for the validation of microarray data is shown in Table 2. Reverse transcription was performed using the Transcriptor First Strand cDNA Synthesis Kit (Roche, Basel, Switzerland); 1 µg of total RNA was used for the process. The quantitative real-time PCR analysis was performed in a Light Cycler 96 (Roche, Basel, Switzerland). SYBER Green I (Roche) was used for the detection of amplified products. All genes were tested in triplicate, and each replicate was on a separate plate (eight biological replicates per group and three technical replicates per mouse). Additionally, each plate contained a series of five-fold dilutions of the cDNA sample to determine the efficiency of the reaction. There were also three negative controls (without cDNA) on the plate. The final reaction volume for each gene was 20 µL. PCR products were subjected to the melting curve analysis using dedicated software for the Light Cycler 96 (Roche, Basel, Switzerland) to confirm amplification specificity. Relative expression was calculated using the Pfaffl method [110]. 

### 4.9. Statistics

The statistical analysis of microarray data is described in Section 4.7 while statistical procedures applied to the remaining data are provided in this section. Corticosterone, glucose and PCR data were first tested for variance homogeneity with C Cochran, Hartley, Bartlett and Levene’s tests. Data that did not meet the requirement of variance homogeneity were first subjected to the square root transformation [111] and next were tested again with C Cochran, Hartley, Bartlett and Levene’s tests. Data with homogenous variance were analyzed with ANOVA followed by the Fisher’s least significance difference (LSD) test. The data that did not meet the requirement of variance homogeneity even after SQRT transformation (corticosterone, water usage, *Sult1a1*, *Lao1* and *Etnppl*) were analyzed with the nonparametric Mann–Whitney U test. Pearson’s coefficient was used to assess the correlation between microarray and PCR results. The data analysis was performed with Statistica software, release 7.1 (StatSoft Inc., Tulsa, USA). Values are presented as mean ± SEM (column bar graphs) and scatter plots.

## 5. Conclusions

Our experiment showed that transcriptomic responses to corticosterone are heterogeneous in terms of the decay latency and that some of them persist for at least 9 h despite a quickly normalized level of blood corticosterone. This indicates that persistent changes in gene expression constitute an important mechanism of the delayed effects of glucocorticoids. Genes that differed more than 50% after 9 h of rest suggest that the affected long-term processes include the metabolism of lipids, ketones and glycogen, homeostasis of iron, water and potassium, regulation of blood pressure, voltage-sensitive chloride channels, actin dynamics, epigenetic modifications, inhibition of tissue remodeling, peroxisomal transport and, finally, the removal of toxins and signaling molecules. On the other hand, a number of transcriptomic responses that display a short-term duration or even time-dependent reversal during the resting period are involved in the negative control of cell growth and proliferation. Therefore, the obtained results suggest that GC-induced impairment in cell growth and proliferation is prone to recovery during resting periods associated with the low level of glucocorticoids. Finally, the obtained results indicate that GCs can contribute up to 63.7% percent of transcriptomic responses observed during the stress response.

## Figures and Tables

**Table 1:** The characteristics of microarrays (SurePrint G3 Mouse Gene Expression v2 8 × 60 K Microarray, 8 × 60 K).

Total number of microarray probes	62,976
Number of technical/control probes	3671
Number of unique probes detecting mouse genes and printed in 1 copy on the microarray	56,305
Number of unique probes detecting mouse genes and printed in 10 copies on the microarray	300
Total number of unique probes detecting mouse genes (without copies of probes)	56,605
Total number of probes detecting mouse genes (including copies of probes)	59,305

**Table 2:** PCR primers.

Gene Name	Forward or Reverse Primer	Primer Sequence	Annealing Temperature	Efficiency
*Sult1a1*	F	GATGGGAAAGTGTCCTATGGGT	60 °C	98.8%
	R	TGAAGGATGTGTGGTGAACAATTA		
*Lao1*	F	ACAACGCTATCGTGCCTCAG	60 °C	95%
	R	CATCAGGTAAGCCTTGGTGGA		
*Etnppl*	F	TTGGTGAAGGACCGTGAGAAA	60 °C	108.6%
	R	AACTTTGCATCGTCTTCCGTG		
*Apoc3*	F	ATGGAACAAGCCTCCAAGACG	60 °C	111.7%
	R	TTGCTCCAGTAGCCTTTCAGG		
*Tbp*	F	GCAGTGCCCAGCATCACTATT	60 °C	108.3%
	R	AAGCCCTGAGCATAAGGTGG		

## Data Availability

The data are available in GEO database (accession number GSE218508) and Supplementary Files.

## Supplementary Materials

The supporting information can be downloaded at: https://www.mdpi.com/article/10.3390/ijms24032828/s1.
